# Multi-Step Polynomial Regression Method to Model and Forecast Malaria Incidence

**DOI:** 10.1371/journal.pone.0004726

**Published:** 2009-03-06

**Authors:** Chandrajit Chatterjee, Ram Rup Sarkar

**Affiliations:** Centre for Cellular and Molecular Biology (CSIR), Hyderabad, India; University of Oxford, United Kingdom

## Abstract

Malaria is one of the most severe problems faced by the world even today. Understanding the causative factors such as age, sex, social factors, environmental variability etc. as well as underlying transmission dynamics of the disease is important for epidemiological research on malaria and its eradication. Thus, development of suitable modeling approach and methodology, based on the available data on the incidence of the disease and other related factors is of utmost importance. In this study, we developed a simple non-linear regression methodology in modeling and forecasting malaria incidence in Chennai city, India, and predicted future disease incidence with high confidence level. We considered three types of data to develop the regression methodology: a longer time series data of Slide Positivity Rates (SPR) of malaria; a smaller time series data (deaths due to *Plasmodium vivax*) of one year; and spatial data (zonal distribution of *P. vivax* deaths) for the city along with the climatic factors, population and previous incidence of the disease. We performed variable selection by simple correlation study, identification of the initial relationship between variables through non-linear curve fitting and used multi-step methods for induction of variables in the non-linear regression analysis along with applied Gauss-Markov models, and ANOVA for testing the prediction, validity and constructing the confidence intervals. The results execute the applicability of our method for different types of data, the autoregressive nature of forecasting, and show high prediction power for both SPR and *P. vivax* deaths, where the one-lag SPR values plays an influential role and proves useful for better prediction. Different climatic factors are identified as playing crucial role on shaping the disease curve. Further, disease incidence at zonal level and the effect of causative factors on different zonal clusters indicate the pattern of malaria prevalence in the city. The study also demonstrates that with excellent models of climatic forecasts readily available, using this method one can predict the disease incidence at long forecasting horizons, with high degree of efficiency and based on such technique a useful early warning system can be developed region wise or nation wise for disease prevention and control activities.

## Introduction

Malaria is one of the major micro parasitic infections causing human mortality in many areas of the world including India. The disease is caused by four species of *Plasmodium* pathogens namely *Plasmodium falciparum*, *Plasmodium vivax*, *Plasmodium ovale*, and *Plasmodium malariae,* and the female *Anopheles* mosquito, one of the most capable vectors of human disease, transmits malaria from one host to another. On an average, malaria infects 300–500 million people and kills 1.5–2.7 million people every year [1], [2]. However, the few resources available indicate that malaria is presently endemic in most of the tropical countries, including Southern America, Asia and the most of Africa, and it is in sub-Saharan Africa where 85–90% of mortality occurs due to malaria [3]. Moreover, in India, malaria is highly endemic in most parts of the country except in areas 5000 feet above the sea level and further, few states namely, Orissa, Uttar Pradesh, Gujarat, West Bengal, Maharashtra, Madhya Pradesh, Rajasthan, Karnataka and Andhra Pradesh account for almost 90% of the malaria incidence in the country [4]. Linking several tools and methodologies related to malaria control into a coherent, integrated approach is extremely challenging even in India. In recent years, analyses of mathematical models and comparisons with incidence data have uncovered fundamental mechanisms, like, dynamics and persistence of parasite infections [5], [6], [7], study of the spatial spread of diseases [8], [9], [10], investigation of underlying recurrent epidemic behavior [11], [12] and further, have been useful for estimating a critical vaccination level, which may eradicate an infection with high success rate under a given set of constraints [13], [14]. Mathematical modeling of malaria has flourished since the days of Ross, who was the first to model the dynamics of malaria transmission [15]. Macdonald has expanded Ross' work introducing the theory of super infection [16]. Since then, efforts have been made to model the malaria incidence using several approaches [17], [18], [19].

Surprisingly, however, despite the current sophistication of the literature, the insights gained from theoretical work have little impact on empirical approaches to epidemiological study and design of public health policy. Therefore, major emphasis must be placed on data oriented studies, though theoretical works play a role in the solution of practical problems in disease control and in the interpretation of observed trends. Further, epidemiological research on micro-parasitic infection such as malaria is largely based on two distinct measures of parasite abundance within communities of people. The first of these is the incidence of infection or disease and the second measure is the prevalence of infection or disease. The measurement of incidence or prevalence is often based on the stratification of the population under study with respect to a variety of factors like age, sex, social factors, environmental variability etc [5], [20], [21]. This indicates the requirement of an alternative modeling approach, namely statistical modeling, based on the available data on the incidence or prevalence of the disease and other related factors. The need of a statistical model primarily arises when numerical output from abstract models needs to be characterized for understanding the behavior of the model and to assess ability for simulating important features in it (a process called *model validation and diagnostic checking*). Further, we need a statistical model to estimate input parameters from complex mathematical models (*parameter estimation and model build up*) and under a non-deterministic system, if we need to predict the future behavior of a system based on past observations, then statistical prediction models provide the best option (*forecasting*).

Significant research in statistical modeling of malaria has been carried out in recent times to gauge the effect of relationship between the disease incidence and climatic factors [22]. However, in some of the previous works in this field, there exist some drawbacks of their own, which may affect the suitability of the model being fitted into the incidence pattern of the disease. For example, lack in continuity of time series at high temporal resolution in the usage of an extensive data set for studying the climatic suitability in malaria transmission in Africa through the *Malaria Risk in Africa* (MARA) project [23] and similarly in Europe by Kuhn et al. [24]. Non-climatic factors like vector abundance, population immunity, etc., have been studied as influential factors for malaria incidence [25] and Hay et al. [26] predicted malaria seasonal pattern in Kenya using Normalized Difference Vegetation Index (NDVI). Bouma et al. [27] in Punjab, India, applied quantitative modeling, but since this model operates at a very low temporal resolution, the robustness of the model is questionable. Further, Chattopadhyay et al. [20] proposed a regression model on *P. falciparum* malaria deaths in Kolkata Corporation, India, considering different environmental as well as social factors and compared their model with a modified version of Ross's model. However, their approach was purely dependent on linear regression after suitable transformation of the selected variables. Very recently, Khanum et al [28] explored the evidence and causality between health and poverty, and illustrated the health-poverty-human security nexus through regression analysis to demonstrate the nexus between malaria risk and poverty. But, the inefficiency of poverty as a single variable cannot explain the status of malaria risk in a country or a region properly. Moreover, the usage of regression analysis for predictions over such a long range as 25 years is probably not a very accurate device for forecasting. The primary climatic factors, such as, temperature and rainfall, have been used in prediction of malaria risk by Ye et al [21] through a binary logistic regression modeling with fractional polynomial transformations. In this approach, the speculation from the induction of linearity between two real life variables (malaria deaths and temperature) through fractional polynomial algorithm is also not obvious for modeling malaria risk with climatic factors. Ruru and Barrios [29] have considered another approach assuming the dependent variable (malaria deaths) as a discrete random variable and modeled malaria risk with a Poisson regression model, which is robust against the stochastic assumptions, which the data need to satisfy. However, due to the skewed nature of response variables, linear regression in this case is not suitable and the problem with the Poisson regression model lies in its basic assumption of discreteness of response variables, which may not be applicable in real situations. Other than such statistical models, in recent studies, researchers have shown seasonality pattern in the malaria incidence with the help of exponentially weighted moving average models (ARIMA) [30] and the Monte-Carlo Markov chain methods [31] with newly developed Bayesian techniques of *a-priori* probability assignment to the risk factors. In our study, we try to deal with these drawbacks and come up with a practical solution.

Considering all the above statistical approaches and subsequent drawbacks, we propose a simple non-linear regression methodology to forecast malaria incidences under natural scenario by considering the effect of environmental factors with no *a-priori* assumptions (which has not been accounted in some of the pervious works) on independent variables and on the nature of response variables (either discrete or continuous). In this study, we not only take into account intuitively the most influential factors, like humidity and temperature, but also try to account for all the other environmental factors, which are significantly important in affecting the incidence pattern of the disease. Further, we do not consider forceful induction of linearity into the model and also consider an unlikely variable, namely, the dependent variable at lag-one in time, which is basically an “auto correlative concept” unlike some of the classical statistical concepts. Thereby, we propose a very lucid approach for modeling malaria incidence in the Chennai city, Tamil Nadu, India, with a simple as well as flexible methodology and with a good capacity of forecasting over both long and short horizons. Initially, we consider a longer time series data of Slide Positivity Rates (SPR, details in Materials and Methods section) values of malaria (both *P. falciparum* and *P. vivax* cases) and try to model the malaria incidence (in the form of SPR values) considering climatic factors, population, along with an additional regressor “SPR values with lag one” as independent variables (since it is quite natural to consider that the incidence of the disease depends on the previous occurrence and persist as well as transmit in the population over time). Due to the inclusion of this one-lag-SPR value as an independent variable, the model give autoregressive forecasts and is useful to predict future incidence with *a-priori* knowledge of previous occurrences of the disease along with the current environmental situations. Secondly, we consider a smaller time series data and try to establish the pattern of incidence over a short period and also of different nature (deaths due to *P. vivax*) for the Chennai city as a whole, based on the influence of the climatic factors and population only. Finally, we introduce another study, which is an approach to map at a finer level (zonal distribution) considering the space as well as the disease incidence. In this case, we study the pattern of malaria incidence in the form of *P. vivax* deaths over the ten constituent zones of the city, separately. In our approach, we not only try to bridge the relation between climatic factors and the malaria incidences, but also look at each zone independently to find out which climatic factors are more influential in which of the zones and to what extent. It is noteworthy, that with advent of excellent multivariate analysis techniques, such as Factor and Cluster Analysis, we significantly reduce the cumbersome demands of the above study after grouping out the environmental factors as well as zonal incidences, which have common underlying pattern or behavior. In this case, we draw general conclusions about the disease pattern over space, relatively on a small scale (restricted in the city level only), and further, predict the disease incidence to suggest better precautionary measures with a better understanding of the pattern and linkage of the disease with climatic factors at the zonal level. It is of considerable importance to note that this combination of multivariate analysis techniques with our basic methodology to model disease incidence at zonal level can be generalized to any volume of data over any spatial range for achieving a great degree of predictability on malaria incidence over time under different environmental factors.

## Materials and Methods

### Collection and organization of data

The data collection was carried out in three phases, namely data on malaria incidences, climatic factors and population. The data on malaria incidence is obtained from the Corporation of Chennai, Chennai, Tamil Nadu, India (zonal division in Supporting Information, Section A of Text S1 and Fig. S1). The data thus obtained is of two types:

- The first data set consists of Slide Positive Rates (SPR) values (for two types of malaria *P. vivax* and *P. falciparum*) over 37 time points in the city of Chennai (as recorded by the Corporation) from January 2002 to January 2005. SPR values are calculated by the equation:
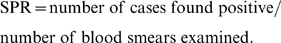

- The second data set consists of the number of *P. vivax* deaths over the ten zones of Chennai city over 12 time points from January–December 2006. The data also has the population for each of 10 zones for each month in the given time period.

We consider population as an additional factor in our study, since, it is very logical to realize that, more the number of people exposed to the disease, the higher will be the number of incidences. The population data is available for the 12 months of the year 2006 for all zones from the Corporation of Chennai. But the population for all the months between January 2002 and January 2005 is obtained [32], [33] through polynomial fitting based on decadal population data from Tamil Nadu census (details in Supporting Information, Section B of Text S1 and Fig. S2). The crude data thus obtained is organized in order to perform suitable statistical analysis. Also, due to the high auto correlation at lag-one, exhibited by the SPR time series, we consider SPR values at lag-one as another independent variable in our study (Supporting Information, Section C of Text S1 and Fig. S3).

Moreover, we consider five environmental factors as regressors, namely, Minimum Temperature, Maximum Temperature, Minimum Humidity, Maximum Humidity, and Total Rainfall. However, due to non-availability of the climatic data from the Corporation of Chennai, we collected those from other sources. Monthly averages of minimum and maximum temperature for January 2002 to January 2005 [34], monthly total rainfall for January 2006 to December, 2006 [35], the monthly averages of total rainfall for the year 2002 from January to December [36] are obtained from the sources, readily available. However, for the years 2003 and 2004 one can only obtain the seasonal totals of rainfall for the four seasons of the year, namely, South–West monsoon (June, July, August, September), North–East monsoon (October, November, December), Winter (January, February), and Hot weather period (March, April, May) [37]. From this, the monthly data is imputed by random number generation and is assigned to the constituent months of the season (details in Supporting Information, Section B of Text S1). We also obtained daily data on minimum and maximum humidity [34] for all the 37 months over the period January 2002 and January 2005, and thereby calculated the monthly averages. The data on minimum and maximum temperature, minimum and maximum humidity for the 12 months between January–December 2006, are obtained from different source [36].

### Variable selection

First, the variables are selected from the complete set of independent variables in order to reduce size of the analytical data set to be dealt with, without compromising the precision of the model and for targeting out the variables, which are likely to be the most influential on the dependent variable. This is done in a step-by-step procedure. First, we consider simple linear regression models between the dependent variable and the independent variables, pair wise one at a time, and select the variable exhibiting the highest coefficient of determination. Then, keeping the first variable selected from the linear model intact, we look at non-linear relationships from the second order models with one new variable from the remaining variables, each at a time. In the second order models, we choose the variables exhibiting highly significant partial *t*-statistic and we continue this process with higher order models (considering higher order non-linearity) until all variables are exhausted or no other variables meet the criterion set above, whichever earlier.

### Identification of the initial relationship between variables

Once the variables are selected, we determine the optimum relationship between the dependent variable and the independent variables (which are selected in the previous step) in a pair wise fashion through the study of scatter diagrams. The word optimum implies the relationship, which has the largest coefficient of determination and the functional forms are proposed accordingly. It is important to note that in our case, we found the polynomial relationships as the most influential in almost all cases but while fitting a polynomial we keep in mind that the marginal increase in precision is to be compromised for safeguarding simplicity of the relation when an increase in the order of the fit does not increase efficiency of the estimation significantly (Supporting Information, Section D of Text S1 and Fig. S4).

### Multi step induction of variables in the non-linear regression analysis (Model refinement)

**Figure 1:**
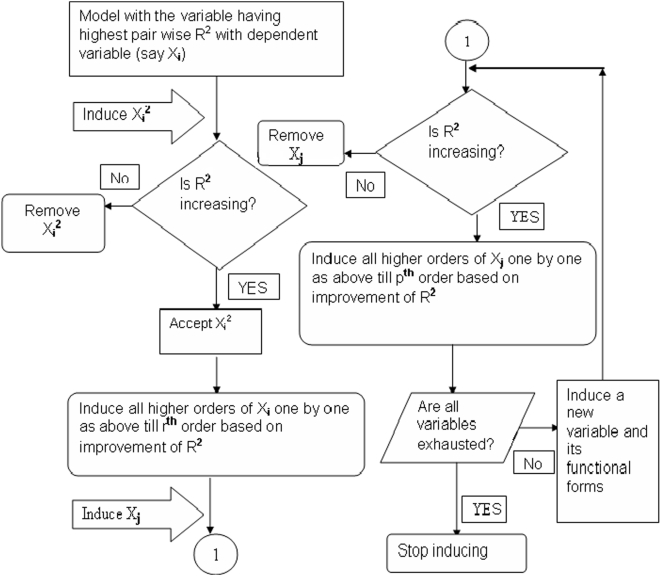
Flowchart for multi step regression model with step-wise induction of variables. X_i_ is the starting variable exhibiting highest coefficient of determination (R^2^) with the dependent variable and the initial pair wise relation is the r^th^ order polynomial, while Xj is the subsequent variable (with p^th^ order relation) with second highest R^2^.

Once the pair wise relationships are identified between selected variables and the dependent variable, we refine the model according to a ‘step wise induction of functional forms and subsequent improvement of residual sum of squares’. The flowchart (Fig. 1) of the process simply explains the procedure and Table S1 (in Supporting Information, Section E of Text S1) denotes the process in detail for the study of SPR values. We start with the variable exhibiting the best pair wise relation with the dependent variable and keep inducing from the lower order functional form of that variable (in case of polynomial function) moving upwards to the higher order depending on the coefficient of determination of the model at each step. When all forms of the first variable are inducted, we then start the procedure again with the functional forms of the second variable in the same way from lower to higher order and repeat the process with other variables until all the selected variables are exhausted.

### Prediction and forecasting through the model

Once we construct the model through the variable selection procedure, identification of initial relationship and multi step induction of variables, we arrive at the model equation. Further, we substitute the values of the independent variables from this equation for each time period to obtain predictions and forecasts for the required time points.

### Testing the correctness of prediction

In our methodology, the final as well as most important step is to test the accuracy of the prediction. Further, it is of utmost importance to test whether the predictions are within an expected range or deviating from the normalcy. Hence, we apply the general Gauss-Markov theory [38] to estimate the confidence intervals for each data points, and find out the degree of confidence to be entrusted for model predictions (detail methodology is in Supporting Information, Section F of Text S1).

### Factor Analysis (FA)

Generally, EFA (Exploratory Factor Analysis) is used to uncover the latent structure of a set of variables [39] and it is a non-dependent procedure, that is, it does not assume any specificity in regards to the dependent variable as such and is used to reduce a larger number of variables into a smaller number of factors (for modeling purposes), where the larger number of variables precludes modeling all measures individually. We perform factor analysis with Principal Component Analysis (PCA) as the method of extraction of factors, which is preferred for purpose of data reduction. In our method of factor analysis we use this Exploratory Factor Analysis (EFA), where the *a-priori* assumption is that any indicator may be associated with any factor. It consists of three steps namely data normalization, factor extraction and rotation of factor axes, where factors are “a new group of variables” formed from the initial dataset that are a linear combination of original variables. We consider this method in our data, since we want to summarize the information contained (rather hidden) in the *P. vivax* deaths over the 10 zones of Chennai city. Our motive is to consider each zone separately and to study which climatic factors affect the P.V. deaths in the ten zones independently. However, instead of proposing ten different models for ten zones, we try to understand the underlying pattern of the zonal deaths grouping together in some pattern according to inter-correlations between the zonal deaths. Then, we proceed with the clustered zones and consider modeling them along with the climatic factors according to the methodology mentioned before.

**Figure 2:**
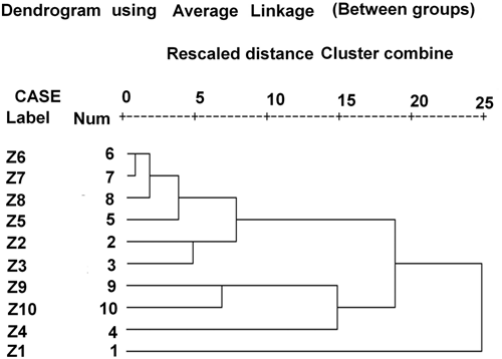
Dendrogram for normalized data set of zonal P. vivax deaths.

In order to reconfirm and cross check the results obtained from FA we also perform a Hierarchical Cluster Analysis (HCA) [40], which has similar concepts of grouping of variables with underlying common behavior. Precisely hierarchical cluster analysis is an unsupervised pattern recognition technique, uncovering intrinsic structure in the data variables. The main aim of HCA is to group objects into classes such that objects are similar inside a class and significantly different from the ones outside that class. Our HCA method starts from the pair of objects, which are most similar and then keep forming higher clusters step by step until all variables in the system are clustered together. This is a typical bottom-up approach of clustering. The results are displayed as a Dendrogram (Fig. 2), which provides a visual summary of clustering process in two dimensions. We use Ward's method [40] (uses an ANOVA approach to evaluate distances between clusters attempting to minimize at each step the sum of squares of any two (hypothetical) clusters that can be formed at each step) on our data set (normalized) to perform cluster analysis.

### Tools used in analysis

MS Excel 2007, MATLAB 7.5.0 [41], and SPSS 11.0 [42] for Windows are used for the various analyses in different parts of our study.

## Results

In the following sections, we discuss the results obtained from our method and model for different types of data series consisting of malaria incidences (SPR and *P.vivax* deaths) over different time periods and zones in Chennai city along with the climatic factors, population and previous incidences.

### 1. Large Time Series

In our first study of Slide Positivity Rates (SPR) values over the period between January, 2002 and January, 2005, the dependent variable is the Slide Positivity Rates in the city of Chennai over a time period of 36 months from January 2002 to December 2004. The regressors are climatic factors as mentioned in the Materials and Methods. However, an initial guess of taking the number of blood smears collected as the dependent variable has been discarded due to the little relationship between the SPR values and the number of blood smears collected (Supporting Information, Section G of Text S1 and Fig. S5). Thus, we consider population as the additional independent factor in our study. As we mentioned before, we consider the SPR values at lag-one as a separate independent variable due to the high auto correlation exhibited by SPR values at lag-one (Supporting Information Section C of Text S1 and Fig. S3). Working as per the method outlined in variable selection methodology, we select four variables, namely, (i) SPR-at-lag-one, (ii) Maximum Temperature, (iii) Minimum Humidity, and, (iv) Population, as independent variables.

Then, we identify the initial relationship through the scatter plots between the dependent variable and the selected independent variables according to the procedure outlined in ‘initial identification of relationships’, and find that the polynomial relationships are the optimum relations between all the pairs (details in Materials and Methods (Section 2)). The scatter plots for this study are shown in Supporting Information, Section D of Text S1 (Fig. S4). Once we find the initial relations, then by the application of the method outlined in model refinement (details in Materials and Methods (Section 2)), we follow the procedure mentioned in the flowchart in Fig. 1 and Table S1 (in Supporting Information, Section E of Text S1), to construct the following model equation:
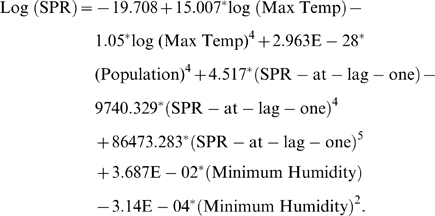
(1)


**Figure 3:**
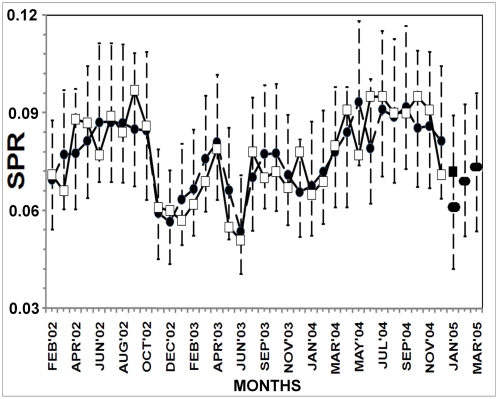
The plot of fit of the predicted values to the observed data of SPR values. Straight line with white squares-observed values of SPR; Dashed line with black circles-predicted values of SPR. Error bars are of 95% confidence intervals of the predicted response. Black ellipses-forecasted values of SPR (autoregressive forecasts) and Black square-observed SPR value for January, 2005.

**Figure 4:**
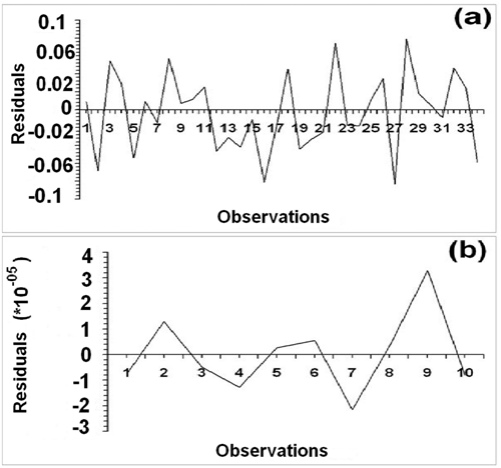
Residual plots for the fitted model. (a) for SPR values and (b) for total P.vivax deaths.

Following equation (1), we predict the values of SPR for the given time points, and, for the months January to March, 2005, we provide the forecasts of SPR values based on the independent variable values (population, climatic factors etc.) of the corresponding time points as well as the SPR values at lag-one. Our forecasted values are autoregressive in the sense that the SPR values of one time point behind are sufficient to provide the forecasts of SPR value of the next time point and each such forecast in turn will act as the model input for the forecast of the successive time points. Along with predictions and forecasts, we also incorporate the 95% confidence intervals as error bars into the plot of fit (Fig. 3) (detail method of confidence interval construction is given in Supporting Information, Section F of Text S1). This error prediction gives a kind of range of variation for the predictions, so that we may infer with 95% confidence the predictions are correct and in our case the predicted responses of the model indicate a good fit overall. Also, the residual plot of the fit for the data (Fig. 4, subfigure (a)) corresponds to no particular pattern over time indicating that the errors tend towards normality. The model yields a coefficient of determination of 63.85% with a highly significant F-value of regression of 6.56 (Table 1).

**Table 1:** ANOVA table of Regression.

Source	Degrees of Freedom (DF)	Sum Squares (SS)	Mean Squares (MS)	F-values (Calculated)	F-values (Tabulated)–at 5% significance level (two-tailed distribution)
**Regression**	8	0.1224	0.0153	6.56	2.32
**Residual**	26	0.0693	0.00267		
**Total**	34	0.1917			

We verify a particular SPR value (January, 2005) with the available data and observe that it matches well with the predicted value and falls in the highly significant confidence region (Fig. 3). We further observe that after the higher incidence of the disease during August–October, 2002 and May–November, 2004, there is again an indication of the increasing trend in the disease pattern starting from January, 2005. Moreover, from the model equation it is clear that SPR-lag-one values have strong influence on the occurrence of the disease (positive effect of fifth order term) and also the population (positive effect of Fourth order term) play important role in the disease incidence. Among the climatic factors, Maximum Temperature and Minimum Humidity play important role to shape the disease curve and this type of effect has also been observed in other studies in different places [43], [44].

### 2. Short Time Series

In our next analysis, regarding the total deaths due to *Plasmodium vivax* (P. V.) in all zones combined, we consider the data set on the first ten time points only, that is, from January to October, 2006 and on the basis of the model thus formed, we forecast the values for November and December, 2006 and match the values with the observed values to justify the predictions. However, in this study we have the yearly population for the Chennai city for all the zones, and hence, we are unable to consider this as a separate independent variable in the monthly regression analysis. So, we consider the population factor through scaling the values of P.V. deaths by the population for the whole city at each time point. Then taking this scaled series as the dependent variable, and following the same procedure of variable selection, we find the following variables as influential - (i) Minimum Temperature, (ii) Maximum Temperature, and, (iii) Maximum Humidity. Following the same procedure as before, we determine the relationships through scatters and enter the functional forms into the final model refinement procedure to reach the model equation as below:
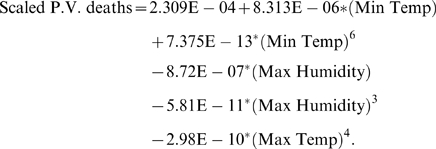
(2)


**Figure 5:**
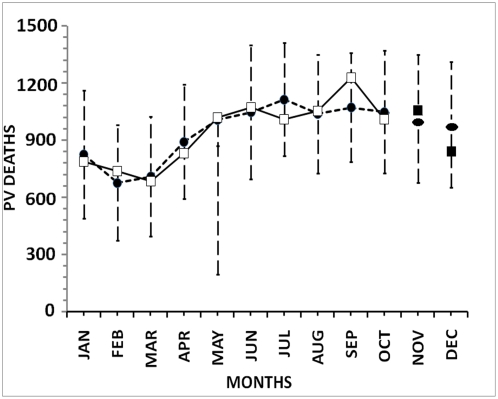
The plot of fit of the predicted values to the observed data of P.vivax Deaths. Straight line with white squares-observed values of P.vivax Deaths; Dashed line with black circles-predicted values of P.vivax Deaths from the model. Error bars are of 95% confidence intervals of the predicted response. Black ellipses-forecasted values of P.vivax Deaths and Black squares-observed values of P.vivax Deaths for November and December, 2006.

From equation (2), we get the scaled values predicted during January–October, 2006. On the basis of this model and values of independent variables incorporated, we also forecast the P.V. death values for the next two months (Fig. 5). Further, we provide the 95% confidence intervals for prediction response in the form of error bars. This model gives a coefficient of determination of 82.6% and an F-value of regression of 3.79, which is significant for a two-tailed F-distribution with (5,4) degrees of freedom at 5% level of significance. Also, the residual plot (Fig. 4, subfigure (b)) shows no particular pattern over time indicating near normality of the error part in the regression.

From this study, we observe that the P.V. deaths show a decreasing trend after remaining almost constant (though high) over the period June–October, 2006. Further, other climatic factors, Minimum Temperature and Maximum Humidity start to play crucial roles along with Maximum Temperature (like before) to shape the disease curve and Minimum Humidity goes out of the model being less influential this time. This dependency of disease incidence on the climatic facotrs also resembles with some other studies in this direction [44], [45]

### 3. Zonal (Spatial) Analysis

Our final analysis is based on zonal analysis of *P. vivax* deaths and proposal of suitable models for each zone considering the effects of environmental factors. For this purpose, we first find the scaled (with respect to population) P.V. deaths for each zone (Materials and Methods) and perform a factor analysis in order to reduce the analytical dimension as mentioned before. Simultaneously, we also study hierarchial cluster analysis (HCA), the results of which are summarized below:

- The Kaiser-Meyer-Olkin measure of sampling adequacy [46] is 0.644 (given Kaiser's recommendation is 0.5), and is a reasonably good measure to suggest that the sample size is good enough to carry forward the analysis.
- Bartlett's test [46] of sphericity, which tests the null hypothesis that the correlation matrix is identity, gives a chi-square value of 65.09, which at 5% level is highly significant for 45 degrees of freedom of the distribution.
- To carry out further analysis, Kaiser's recommendation [46] is that the determinant of the correlation matrix of variables under study should be greater than 1×10^−05^. However, in our case it is 7.297×10^−05^, which indicates it is good enough to carry out the process.
- From our analysis, we find that three factors have Eigen values greater than 1 as demonstrated by the Scree plot (Supporting Information, Section G of Text S1, Fig. S6). However, we consider 6 factors to ensure that the cumulative variance explained by the factors exceeds 90%.
- Next, we search for the Communalities [47], which are the measures of significant variance of a particular variable to be described by a particular set of factors. For a good factor analysis, the number of factors should be less, and communalities high (close to 1), and the factors are readily interpretable in terms of particular sources or process. In our case, from the factor analysis we find 6 factors are influential to be reproduced to a large extent with very high communalities, idealizing the logic behind carrying out factor analysis.
- Moreover, we study the Factor loadings, which show how the factors characterize the variables. High factor loadings (close to 1 or −1) indicate strong relationship between the variables and the factor describing the variables. The factor loadings matrix is then rotated to an orthogonal simple structure (Section G of Text S1 and Table S2) according to Varimax Rotation Technique [47] justifying our claims.

Then, we perform the HCA using Ward's method [47] in order to visualize the unsupervised pattern and recognize as well as uncover the intrinsic structure of the data without making *a-priori* assumption about the data. The Dendrogram (Fig. 2), in this context gives an excellent and interpretable view of underlying pattern of variables. From these analyses, we cluster out 5 groupings of zones, which have common behavioral pattern and intrinsic underlying structure namely: *Cluster I:* Scaled deaths in Zones 5, 6, 7, 8; *Cluster II:* Scaled deaths in Zones 2 and 3; *Cluster III:* Scaled deaths in Zones 9 and 10; *Cluster IV:* Scaled deaths in Zone 4; and *Cluster V:* Scaled deaths in Zone 1.

**Figure 6:**
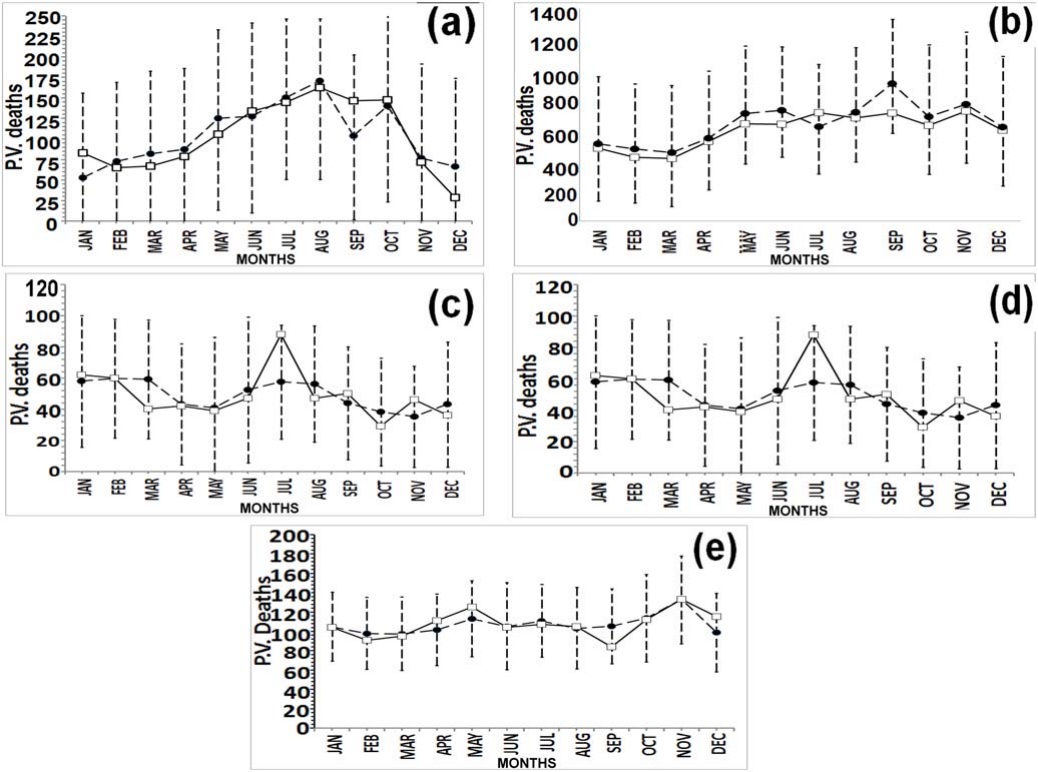
Plot of fits of predictions based on the proposed model against the observed values for 5 clusters. (a) cluster I, (b) cluster II, (c) cluster III, (d) cluster IV, (e) cluster V (Straight line with white squares-observed P.vivax Deaths; Dashed line with black circles-predicted values of P.vivax Deaths from the model). Error bars are of 95% confidence intervals of the predicted response.

Next, we model each cluster with respect to all the other climate variables. We state the final variables selected and the equation obtained for each cluster in Table 2. Fig. 6 shows the plot of fit as well as the predictions based on our model for each cluster. The residual plots of the models for each cluster are shown in Supporting Information (Section G of Text S1, Fig. S7).

**Table 2:** The final model fits for individual clusters.

CLUSTERS	Variables selected	Model Equations proposed for the clusters (Y = scaled P.V. deaths)	Coefficient of Determination (R^2^) and ANOVA Test
**I**	Minimum Temperature (x_1_), Maximum Humidity (x_2_), Total Rainfall (x_3_),	Y = 8.19E-04+2.73E-05 x_1_−1.55E-08 x_1_^3^+8.638E-06 x_2_−5.43E-12 x_2_^4^−3.9E-07 x_3_+2.859E-09 x_3_^2^−1.39E-14 x_3_^4^	R^2^ = 75.03%F-value: 1.71d.f.: (7, 4)Sig.-F: 0.31*
**II**	Minimum Temperature (x_1_), Total Rainfall (x_3_), Maximum Temperature (x_5_)	Y = 4.899E-03−5.34E-04 x_1_+1.174E-05 x_1_^2^+ 3.992E-06 x_3_−2.53E-08 x_3_^2^+4.637E-11 x_3_^3^+ 5.177E-06 x_5_^2^−1.16E-07 x_5_^3^	R^2^ = 77.2%F-value: 1.93d.f.: (7, 4)Sig.-F: 0.27*
**III**	Minimum Humidity (x_4_), Maximum Temperature (x_5_)	Y = −3.08E-04+8.628E-06 x_4_−8.658E-08 x_4_^2^+7.613E-06 x_5_−1.90E-12 x_5_^5^	R^2^ = 34.2%F-value: 0.91d.f.: (4, 7)Sig.-F: 0.51*
**IV**	Maximum Humidity (x_2_), Total Rainfall (x_3_), Minimum Humidity (x_4_),	Y = 5.958E-04−3.64E-06 x_2_−4E-07 x_3_+1.533E-10 x_3_^2^+3.913E-18 x_3_^5^−1.07E-07 x_4_^2^+2.318E-13 x_4_^5^	R^2^ = 69.59%F-value: 1.91d.f.: (6, 5)Sig.-F: 0.25*
**V**	Total Rainfall (x_3_), Minimum Humidity (x_4_),	Y = −1.14E-03+1.861E-06 x_3_−2.08E-08 x_3_^2^+6.552E-11 x_3_^3^−1.48E-16 x_3_^5^+4.413E-05 x_4_−3.49E-07 x_4_^2^	R^2^ = 50.34%F-value: 0.84d.f.: (6, 5)Sig.-F: 0.58*

* The ANOVA of regression yields the F-values, which are significant at the 5% level of significance for corresponding degrees of freedoms of the F-distribution.

The fitted model and the observed disease incidence for the zonal clusters reveal some interesting features, which are not observed in the model for total incidences considering all the zones together. In the model of combined zones, we observe a decreasing trend of the P.V. deaths similar in some of the cluster models (Cluster-I and II) but surprisingly other clusters (III, IV and V) show increasing trend of the deaths. Though there are different combinations of climatic factors (not all same) affecting the death incidence in different clusters (Table 2), interestingly Total Rainfall comes to play crucial role for the first time in the model for the clusters I, II, IV and V, though it is not observed in the model of total deaths in all zones combined. Further, the death incidences predicted and observed in different clusters indicate the similarity in the pattern of geographical as well as social nature of corresponding zones, and hence, increasing trend of P.V. deaths reflecting out from cluster IV (zone 4) and cluster V (zone 1) is quite natural in this context.

It is important to note that, the model, being a step-wise model, determines the parameters through an optimization procedure (optimization being with respect to the residual sum of squares at each step in the model). Parameters are not inputted at the beginning of model, but throughout the process of model construction, we define, choose, and refine the parameters. Hence, the model refinement takes care of possible combinations of parameter inputs for a particular model and ultimately chooses the parameters and their particular functional forms, which are most influential in that particular incidence pattern. The model being specific to the study of a particular incidence pattern, once the model is presented there is no need of re-parameterization. Moreover, the current model is robust to stochastic and sublime changes that may arise due to anomalies as epidemical outbreaks of the disease.

## Discussion

Malaria is one of the most severe problems faced by the tropical countries, specifically Indian subcontinent and Africa [4], [48]. The problems of unavailability of a single accepted control measure coupled with increased drug resistance of the parasite causing malaria have made it even more lethal. However, there are projects being undertaken all over the world by international organizations to find a way out to tackle the menace [24]. In a broad sense, there are two major approaches towards tackling the malaria risk. One method involves the control of the parasite through the control of vector on a geographical basis. The other is to understand the causative factors and underlying transmission mechanics of the disease and on basis of this *a-priori* information one can map and predict the risk of disease in terms of the causative factors [21], [28], [30], [31], [43], [44].

We primarily study this second approach and concern with the effects of climatic factors exerted on the malaria incidence in a particular area. In this paper, we model the malaria incidence on the basis of three types of data – a large time series spanned over 36 months, a short time series spanned over 12 months and a short time series but with wider analytical scope (zonal distribution of *Plasmodium vivax* deaths over the ten zones of Chennai) along with various climatic factors. The earlier works specific to this field have concentrated mainly on inducing linearity between variables thereby fitting a general linear model to different influential factors [20], [28] and those who have considered non-linear models, have predefined the functional form of the model before getting into the dynamics between the variables [21], [29]–[31], [49]. However, we consider a route different from this and chose a realistic, simple, highly organized non-linear regression method derived through a step-by-step multiple regression process and also with high predictive power of malaria incidence. We need not predetermine the functional form of the model in its outset, as it is determined in the process through the understanding of the behavior of the underlying relationship of each variable with the incidence of malaria. This also ensures the applicability of the methodology and model to any type of data set and disease situation (outbreak or normal) and needs no prior assumption of parameter values. The results establish our methodology in the sense that, it has demonstrated executability of our method both for short and long time series of data, where normality may prevail for one case and not for the other. It also shows that our model possesses an equal degree of prediction power for two types of malaria incidences namely the Slide Positivity Rates (SPR) and *P. vivax* deaths. In combination with multivariate data reduction processes (like Factor Analysis) considered in this study, this methodology proves to be highly useful in forecasting malaria incidences in a larger spatial scale. Moreover, the results also show the autoregressive nature of forecasting in the long-range time series study, where the one-lag SPR values play an influential role and can be useful for better prediction. We further identify some interesting effects of the climatic factors on the disease dynamics. Temperature and Humidity (both Maximum and Minimum) are the key factors, which play crucial role in shaping the disease curve in all the three types of data. But interestingly Total Rainfall remains influential only for few zones, which came out from the cluster analysis. The disease incidence (specifically, deaths) at zonal level and the effect of different causative factors on different zonal clusters indicate the pattern of malaria prevalence in the city and is one of the important outcome of our analysis. It is interesting to observe different combination of climatic factors affecting the deaths in different ways for different clusters. In addition, the study reveals the similarity in the occurrence of the disease in different zones according to the nature of geographical and social pattern of corresponding zones. Hence, the regression model for each clustered zones and corresponding predictions act as an important indicator for future occurrence of the disease considering the zonal climatic factors as well as other factors.

The study demonstrates that with excellent models of climatic forecasts readily available, one can predict the disease incidence at long forecasting horizons, using this method, with high degree of efficiency. The major advantage of this method is that we do not need any prior assumptions about the disease or knowledge of parameters, only the previous occurrence of the disease and suitable climatic factors are required to feed into the method and consequently better predictions about the disease incidence can be obtained, which is also clear from our case study. It is of considerable importance to note that this can be generalized to any volume of data over any spatial range to achieve a great degree of predictability of malaria incidence over time with respect to the factors considered in the respective study. Further, the model is completely independent of disease state fluctuations as an epidemic or a sudden downfall of the incidence as a post-ad-hoc control strategy. The model, on the other hand, following step-wise methods determines the parameters through an optimization procedure (with respect to the residual sum of squares at each step in the model) and the model refinement process takes care of possible combinations of parameter inputs thereby choosing the most influential parameters and corresponding functional forms. Hence, the model being specific to the study of a particular incidence pattern, once the model is presented there is no need of re-parameterization. Therefore, based on such technique a useful early warning system can be developed region wise or nation wise for disease prevention and control activities.

## Supporting Information

Text S1 Zonal division; Computation of monthly population from yearly data; Computation of monthly rainfall from seasonal total; Correlogram of SPR; Determination of the initial relations between the selected variables and the dependent variable; Multiple regression method for model refinement; Testing the predictions (construction of confidence intervals); Relationship between the SPR values and the number of blood smears collected; Rotated component matrix and Scree plot of the zonal analysis of deaths due to P. vivax; Residual plots of the individual models of Five Clusters
(0.05 MB DOC)

Figure S1 Zonal division of the corporation of Chennai city, Tamil Nadu, India.
(9.31 MB TIF)

Figure S2 Population data for Chennai and fitted population series (Solid lines with squares-observed series; dashed line-fitted polynomial).
(3.63 MB TIF)

Figure S3 Correlogram of SPR values.
(4.12 MB TIF)

Figure S4 Scatter plots between the selected variables and the dependent variables showing the initial functional relationship: (a) log (SPR) vs. log (Max. Temp) - 4th order polynomial; (b) log (SPR) vs. SPR-at-lag-one - 5th order polynomial; (c) log (SPR) vs. Min. Humidity - 3rd order polynomial; (d) log (SPR) vs. Population - 4th order polynomial. These functional forms represent the optimum relationships (highest R^2^ values).
(4.97 MB TIF)

Figure S5 Scatter plot between the SPR and number of blood smears collected.
(3.23 MB TIF)

Figure S6 Scree plot showing the number of factors to be retained.
(2.04 MB TIF)

Figure S7 Residual plots of clusters: (a) Cluster-I, (b) Cluster-II, (c) Cluster-III, (d) Cluster-IV, (e) Cluster-V.
(4.28 MB TIF)

Table S1 Refinement of model through multi step variable induction and improvement of R^2^
(0.03 MB DOC)

Table S2 Rotated component matrix (Varimax Rotation Technique).
(0.03 MB DOC)
