# Supplementary material for: Multi-Step Polynomial Regression Method to Model and Forecast Malaria Incidence
Source: PLoS One. 2009 Mar 6;4(3):e4726. doi: 10.1371/journal.pone.0004726 (PMC2648889; doi:10.1371/journal.pone.0004726)
Supplement: Text S1 — Zonal division; Computation of monthly population from yearly data; Computation of monthly rainfall from seasonal total; Correlogram of SPR; Determination of the initial relations between the selected variables and the dependent variable; Multiple regression method for model refinement; Testing the predictions (construction of confidence intervals); Relationship between the SPR values and the number of blood smears collected; Rotated component matrix and Scree plot of the zonal analysis of deaths due to P. vivax; Residual plots of the individual models of Five Clusters (0.05 MB DOC) [file pone.0004726.s001.doc]

# Text S1

### Section A

**Zonal Division:** Chennai (13◦ 5’ 24’’ N; 80◦ 16’ 12’’ E) city, Tamil Nadu, India, has 10 zonal divisions in accordance with the administrative demarcations of the Corporation of Chennai. The zones are divided according to the regional specifications of the administrative wards of the municipal corporations (Fig. S1).

### Section B

**Computation of monthly population from yearly data:** We obtain the Population Data (census data) of Chennai city at ten years intervals from 1901-2001 and also for 2006 (recorded by Chennai Corporation). To get the monthly population data during the period January 2002 - January 2005, we fit a 3^rd^ order polynomial curve (coefficient of determination (R^2^) of 99.5%) to the yearly data (Fig. S2). More specifically, if x be the time points and y be the corresponding population values then the equation of 3^rd^ order is of the form:

Y = - 4762.3 x^3^ + 126850 x^2^ – 526627 x + 1000000, (SB-1)

From the above equation, we then interpolate the required monthly population for the time points Jan’ 02 - Mar’05.

**Computation of monthly rainfall from seasonal total for the years 2003 and 2004:** We obtain the Rainfall Data in the form of seasonal totals for the years 2003-2004 for the four seasons of the year, namely, South-West monsoon (June, July, August, September); North-East monsoon (October, November, December); Winter (January, February); and Hot weather period (March, April, May). Since, for our analysis, we need the monthly rainfall data, we follow a simple scheme to generate the required data using random numbers for each month separately. First, we consider a particular season (say, South-West monsoon period with 4 constituent months), then we generate 4 random numbers from a uniform distribution in the interval (0, 1) using simple computer program. Further, we find the total of these 4 random numbers and divide each number by the total respectively, to get 4 new (random) numbers that add up to 1 (this is nothing but a simple scaling technique). Then, we multiply these numbers with the corresponding seasonal total to get the monthly rainfall values and assign one of these values to the constituent months of the season. We repeat the same technique for the remaining seasons and subsequently for the remaining years, to get all monthly values of rainfall, which initially are not available in our raw data.

### Section C

**Correlogram of SPR:** To study the autocorrelation function (1) at various lags of a time series, we plot the Correlogram against each lag at which the function is computed. If in a time series *X_t_*, the auto covariance function at *lag k* is defined as *γ_k_= cov (X_t_, X_t-k_)*, where the ‘cov’ denotes simple covariance between the two series, then auto correlation (*ρ_k_*) at *lag k* is defined as,

**ρ_k_= γ**_k_ / **γ**_0_, (SC-1)

where, *γ_0_* denotes covariance at *lag 0* or simply the variance of the series. In our study, we find that there is a high correlation between the SPR values and SPR values at lag one, which indicates the presence of a strong positive relationship between the two series and justifies our consideration of the “SPR-at-lag-one” as an independent variable in the model (Fig. S3).

# Section D

Determination of the initial relations between the selected variables and the dependent variable: To determine the initial functional relationship, we first plot the scatter plots of the selected and dependent variables after suitable transformations (Fig. S4 a-d) and propose proper functional forms which fits better (with highest R^2^ values).

# Section E

**Multiple regression method for model refinement:** In Table S1, we explain the step by step multiple regression method for model refinement and clearly state the study of SPR value-model at each step through the process of model refinement by improvement of residual sum of squares. Here, the variables are denoted as X_1_- log (Maximum Temperature), X_2_**-** Population, X_3_- SPR-at-lag-one, X_4_- Minimum Humidity, Y- log (SPR). At each step we are concerned with improvement of the R^2^ of the model and we accept only those functional forms, which increase the coefficient of determination for the model.

### Section F

**Testing the predictions (construction of confidence intervals):** Once we propose the model after refinement, it gives predictions on the dependent variable, and then we need to check for testing the correctness of these predictions. We construct the 95% confidence intervals for each prediction response of the model following the scheme described below:

Let, the data of the dependent variable consists of *m* observations, *y_1_, …, y_m_*. The dependent variable has an inherent error related to it. This error is assumed to be a zero mean white noise, i.e. a normal random variable with mean zero. Let, there be *n* independent variables *X_1_, …,X_n_* (which are considered to be error free). Then, we have *n* coefficients of regression or *n* parameters; say *β_1_,…,βn*. Hence, if the model is supposed to be of the form

$y_{i}=\sum_{j=1}^{n} X_{ij}\beta_{j}+\varepsilon_{i},$ (SF-1)

where, *ε*_i_’s are *N (0, σ^2^)* (random errors); *i = 1 (1) m*. Further, let us consider the residuals defined as *r_i_* = (observed *y_i_*- predicted *y_i_*). The objective function is defined by *S* = sum of squared residuals. Subsequently, the fit is obtained when this *S* is minimized. Based on certain constraints, the parameters then have minimum variance (following **Gauss-Markov theorem**, (1)) and may also represent the maximum likelihood solution to the optimization of the objective function-problem. Further, from the theory of linear least squares we have:

$\hat{\beta}=(X^{T}X)^{-1}X^{T}y.$If *X* be the matrix formed from the values of the given independent variables ((*X_ij_*)); *i=1(1)m***,** *j=1(1)n*, then the estimated coefficient vector is given as,

(SF-2)

An unbiased estimate of σ^2^ is then given by,

$$\hat{\sigma}^{2}=\frac{S}{m-n}.$$

(SF-3)

Here, *(S/σ^2^) ~ χ^2^_(m-n)_* ( a chi-square distribution with *(m-n)* degrees of freedom).

Now, given the values of the independent variable *X_dj_* the corresponding value of the dependent variable may be calculated as,

(SF-4)

Further, writing the values *X_dj_* in vector form and denoting as *Z*, the *100(1-**α) %* C.I. of the prediction response is given by,

(SF-5)

where, the symbol “*t”* represents the upper -(α/2) ^th^ point for *t*-distribution with *(m-n)* degrees of freedom.

# Section G

**Relationship between the SPR values and the number of blood smears collected:** In our study of SPR values (as dependent variable), initially we have considered the number of blood smears collected as an independent variable, but we observe a poor relationship between these two (shown in the scatter plot in Fig. S5). Hence, we reject the variable “number of blood smears collected” from our study and consider the population as an independent variable (since intuitively also the burden exerted by the existing population on the incidence of the disease cannot be ignored).

**Rotated component matrix and Scree plot of the zonal analysis of deaths due to *P. vivax*:** In factor analysis, through the rotated component matrix, we reach the conclusion for further consideration of the number of factors, and we come to know about particular variables, which load onto the factor and from the Scree plot (Fig. S6) we can find out the number of principal components need to be considered (Kaiser’s recommendation (2) being all having Eigen values greater than 1). Also, the interpretability of factors can be improved through rotation. Rotation maximizes the loading of each variable on one of the extracted factors minimizing the loading of all other factors. Rotation works through changing absolute values of variables whilst keeping their differential values constant. Among different orthogonal rotation techniques such as, Varimax, Quartimax and Equamax, we adopt the *Varimax Rotation Technique* for our study. Table S2 shows the rotated component matrix of our analysis and ensure that these two results match well to justify our claim.

**Residual plots of the individual models of Five Clusters:** We plot the residuals for each model of five clusters in Fig. S7 and it shows that there are lacks of particular patterns in the residual plots respectively, which further imply that the error part of regression follow normality.

**References**

1. Rao C.R. (1972) Linear statistical inference and its applications. India: John Wiley and Sons.
2. Field A (2004) Factor Analysis using SPSS- Discovering Statistics using SPSS. London: Sage.
