# Supplementary figures and images for: Multi-Step Polynomial Regression Method to Model and Forecast Malaria Incidence
Source: PLoS One. 2009 Mar 6;4(3):e4726. doi: 10.1371/journal.pone.0004726 (PMC2648889; doi:10.1371/journal.pone.0004726)

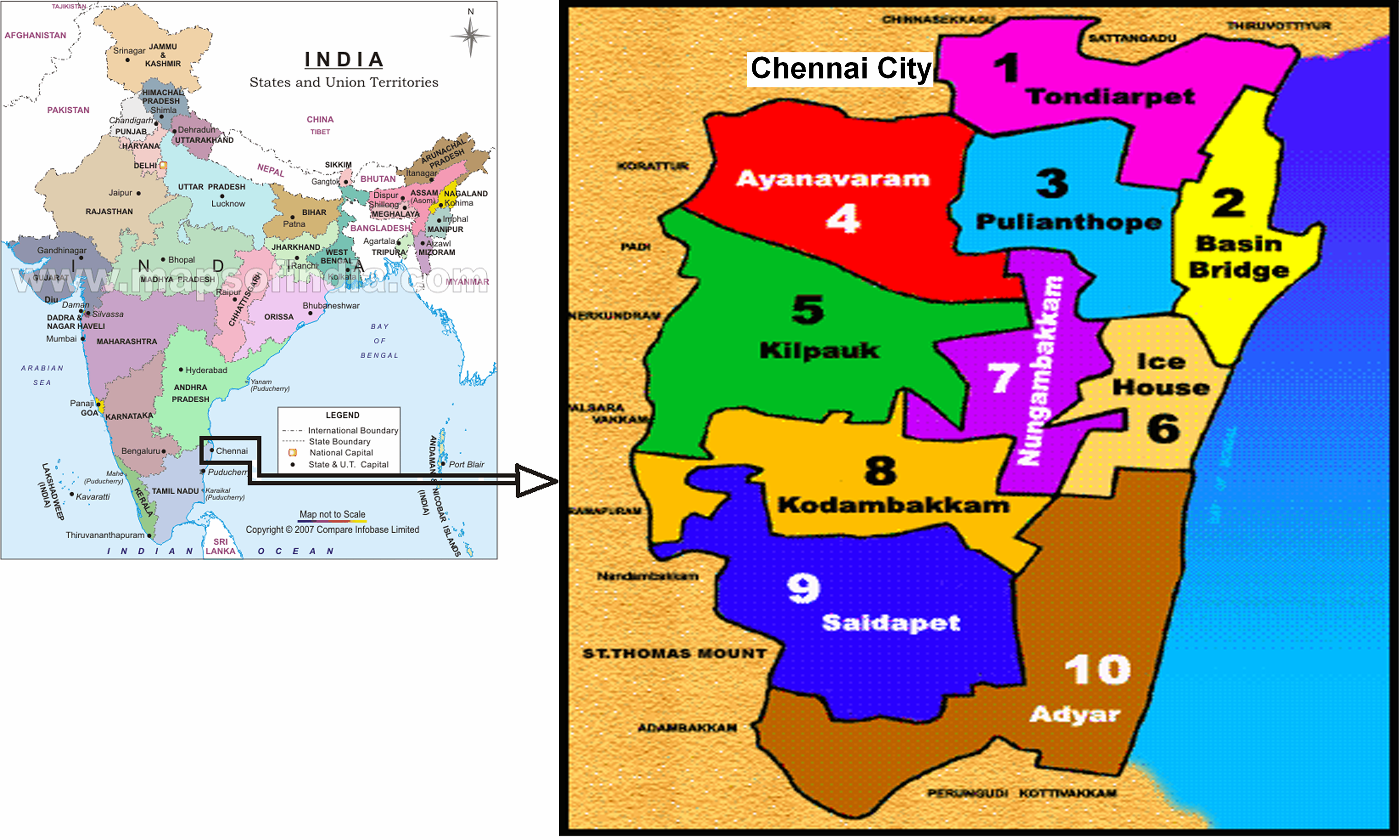

Supplement: Figure S1 — Zonal division of the corporation of Chennai city, Tamil Nadu, India. (9.31 MB TIF) [file pone.0004726.s002.tif]

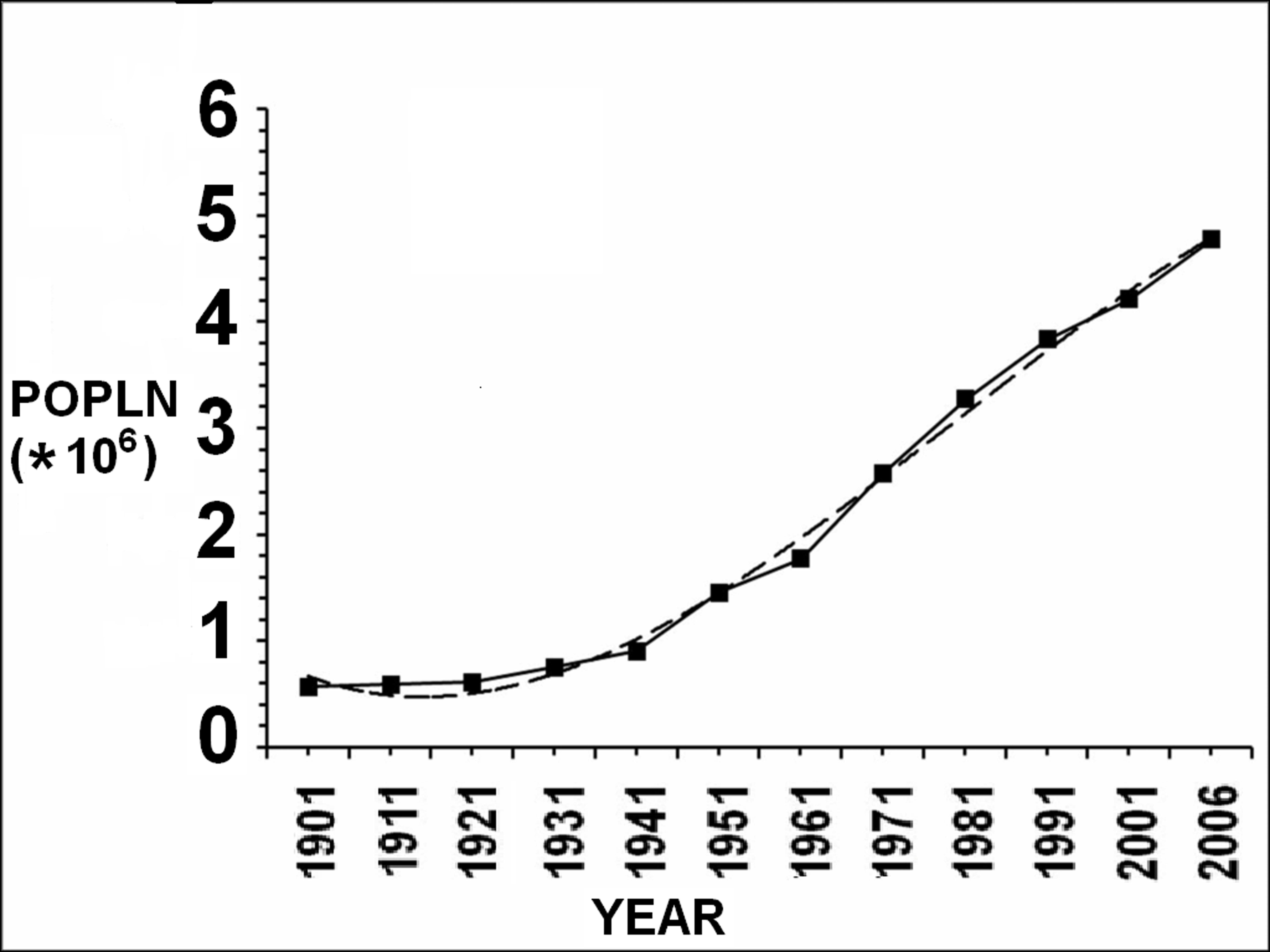

Supplement: Figure S2 — Population data for Chennai and fitted population series (Solid lines with squares-observed series; dashed line-fitted polynomial). (3.63 MB TIF) [file pone.0004726.s003.tif]

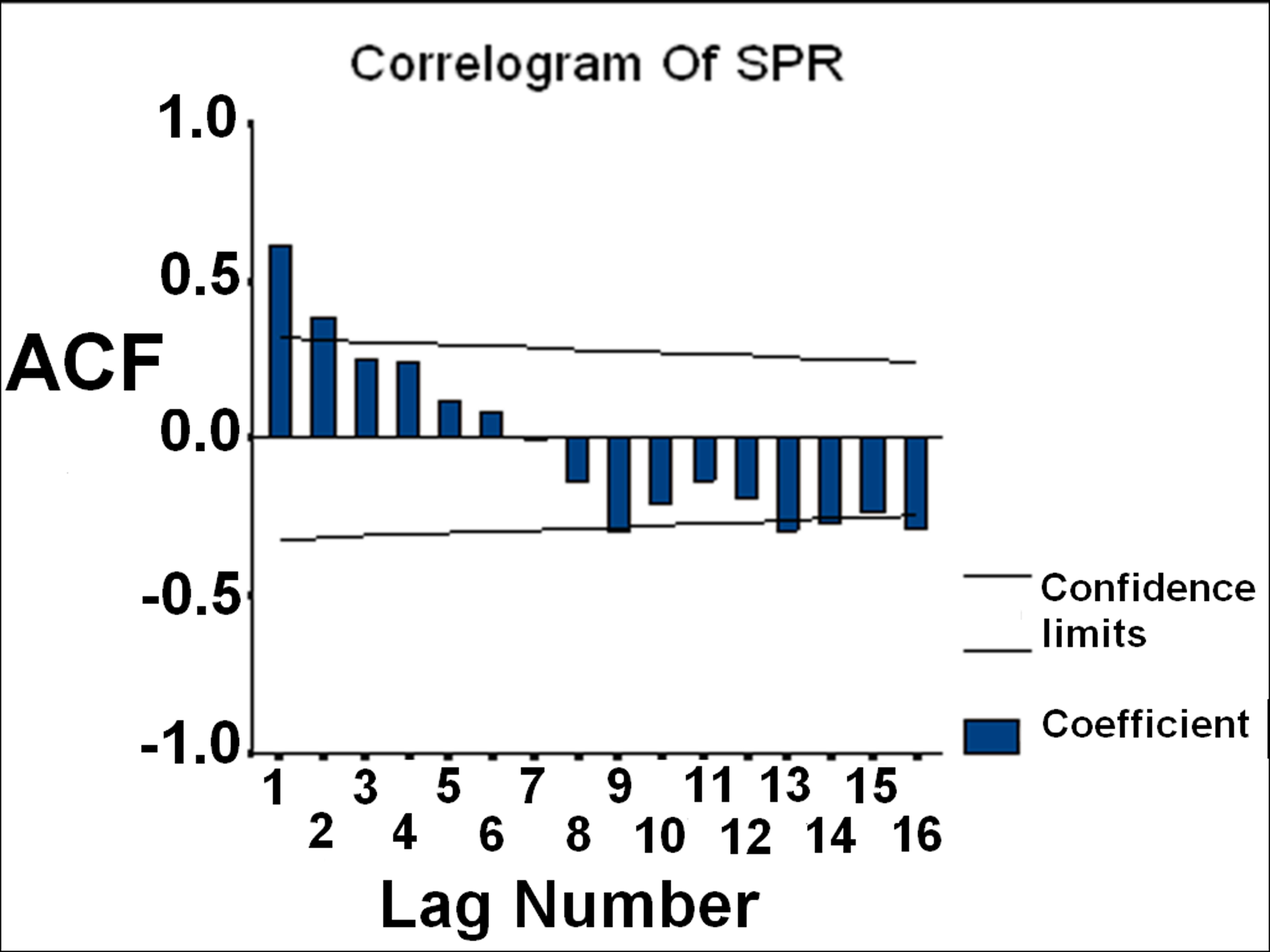

Supplement: Figure S3 — Correlogram of SPR values. (4.12 MB TIF) [file pone.0004726.s004.tif]

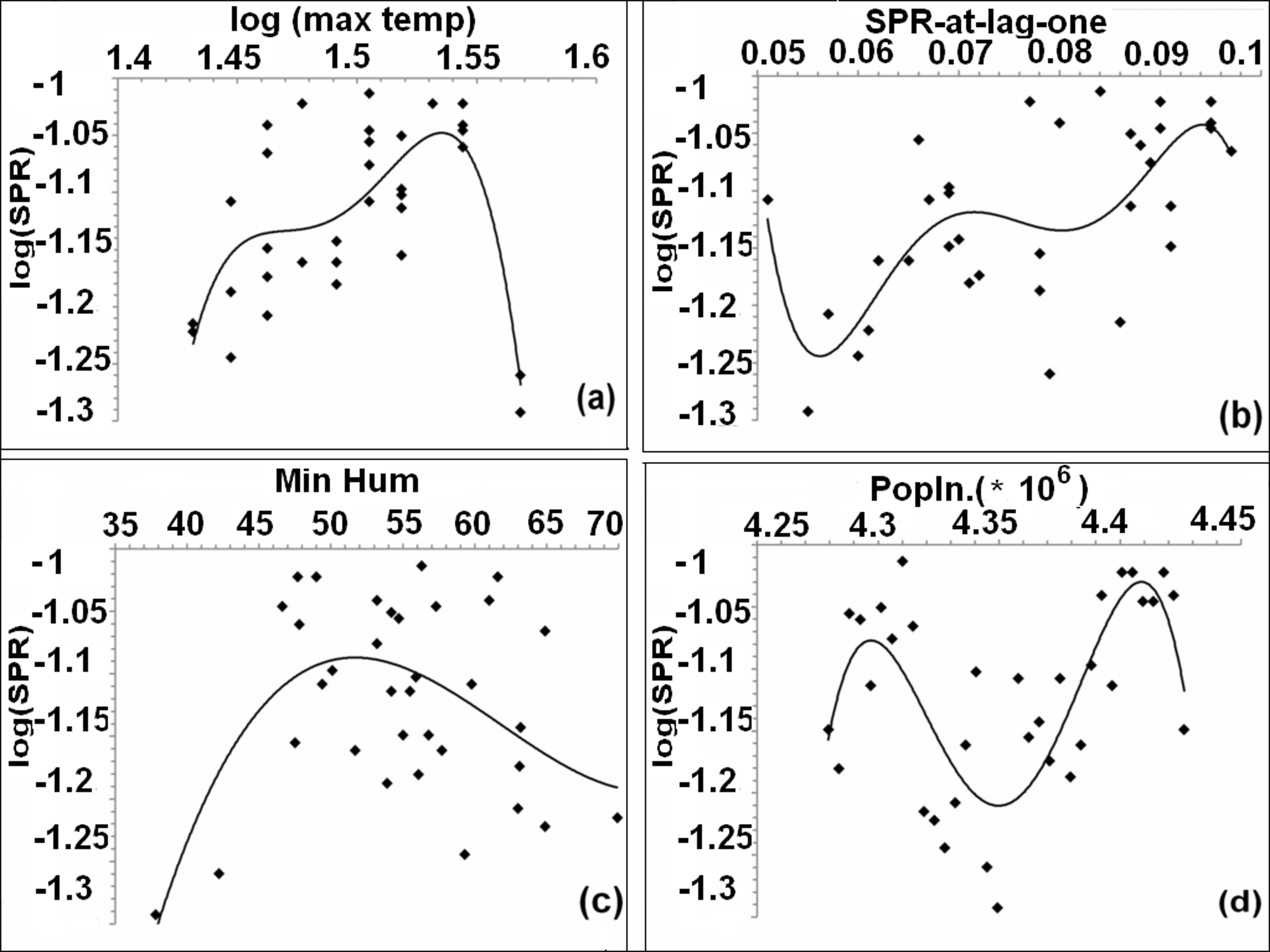

Supplement: Figure S4 — Scatter plots between the selected variables and the dependent variables showing the initial functional relationship: (a) log (SPR) vs. log (Max. Temp) - 4th order polynomial; (b) log (SPR) vs. SPR-at-lag-one - 5th order polynomial; (c) log (SPR) vs. Min. Humidity - 3rd order polynomial; (d) log (SPR) vs. Population - 4th order polynomial. These functional forms represent the optimum relationships (highest R2 values). (4.97 MB TIF) [file pone.0004726.s005.tif]

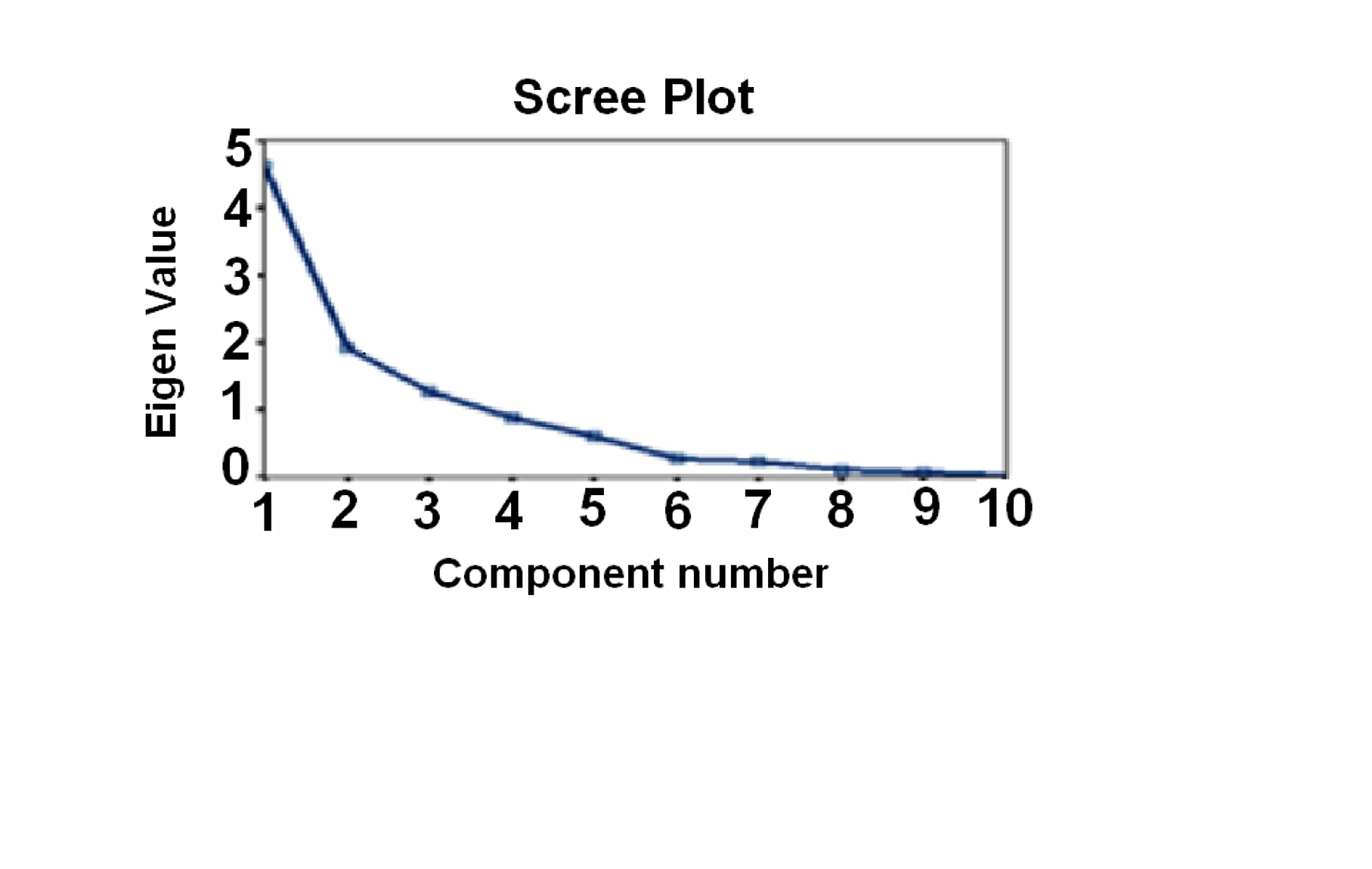

Supplement: Figure S6 — Scree plot showing the number of factors to be retained. (2.04 MB TIF) [file pone.0004726.s007.tif]

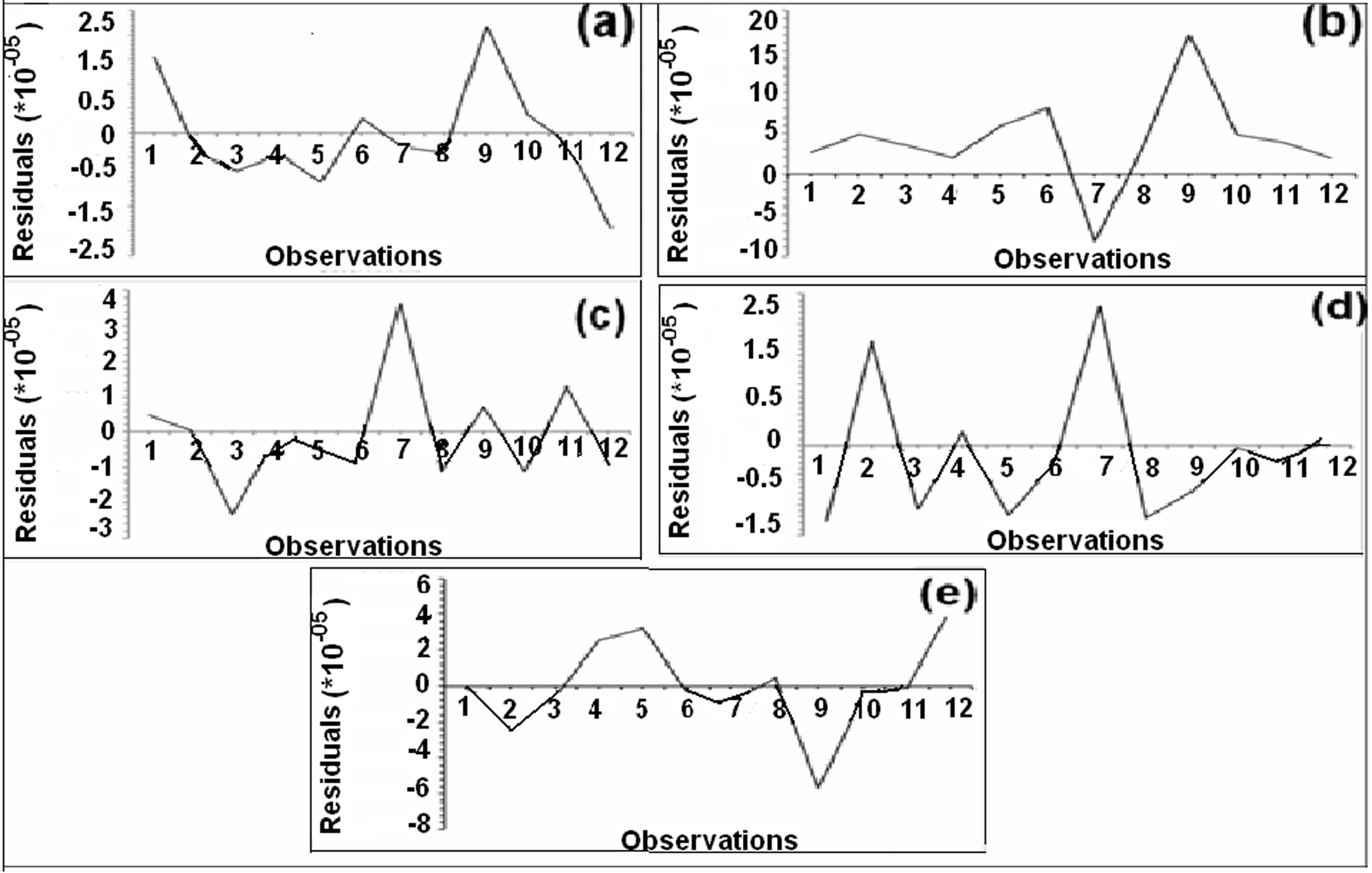

Supplement: Figure S7 — Residual plots of clusters: (a) Cluster-I, (b) Cluster-II, (c) Cluster-III, (d) Cluster-IV, (e) Cluster-V. (4.28 MB TIF) [file pone.0004726.s008.tif]
