# Supplementary material for: Multi-Step Polynomial Regression Method to Model and Forecast Malaria Incidence
Source: PLoS One. 2009 Mar 6;4(3):e4726. doi: 10.1371/journal.pone.0004726 (PMC2648889; doi:10.1371/journal.pone.0004726)
Supplement: Figure S5 — Scatter plot between the SPR and number of blood smears collected. (3.23 MB TIF) [file pone.0004726.s006.doc]

***Table S1: Refinement of model through multi step variable induction and improvement of R^2^***

| **VARIABLE ENTERED** | **ACCEPTED/REMOVED** | **R^2^ OF THE CURRENT MODEL** |
| --- | --- | --- |
| X**_1_** |  | 0.066 |
| X**_1_,** X**_1_^2^** | ACCEPTED | 0.365 |
| X**_1_,** X**_1_^2^**, X**_1_^3^** | X**_1_^2^** REMOVED | 0.368 |
| X**_1_**, X**_1_^3^**, X**_1_^4^** | X**_1_^3^** REMOVED | 0.371 |
| X**_1_**, X**_1_^4^**, X**_2_** | ACCEPTED | 0.418 |
| X**_1_**, X**_1_^4^**, X**_2_**, X**_2_^2^** | X**_2_** REMOVED | 0.419 |
| X**_1_**, X**_1_^4^**, X**_2_^2^**, X**_2_^3^** | X**_2_^2^** REMOVED | 0.419 |
| X**_1_**, X**_1_^4^**, X**_2_^3^**, X**_2_^4^** | X**_2_^3^** REMOVED | 0.42 |
| X**_1_**, X**_1_^4^**, X**_2_^4^**, X**_3_** | ACCEPTED | 0.603 |
| X**_1_**, X**_1_^4^**, X**_2_^4^**, X**_3_**, X**_3_^2^** | ACCEPTED | 0.604 |
| X**_1_**, X**_1_^4^**, X**_2_^4^**, X**_3_**, X**_3_^2^**, X**_3_^3^** | X**_3_^2^** REMOVED | 0.604 |
| X**_1_**, X**_1_^4^**, X**_2_^4^**, X**_3_**, X**_3_^3^**, X**_3_^4^**, X**_3_^5^** | X**_3_^3^** REMOVED | 0.607 |
| X**_1_**, X**_1_^4^**, X**_2_^4^**, X**_3_**, X**_3_^4^**, X**_3_^5^**, X**_4_** | ACCEPTED | 0.612 |
| X**_1_**, X**_1_^4^**, X**_2_^4^**, X**_3_**, X**_3_^4^**, X**_3_^5^**, X**_4_**, X**_4_^2^** | ACCEPTED | 0.638 |
| X**_1_**, X**_1_^4^**, X**_2_^4^**, X**_3_**, X**_3_^4^**, X**_3_^5^**, X**_4_**, X**_4_^2^**, X**_4_^3^** | REJECTED | 0.636 |
| **NO MORE INDUCTION,**  **ACCEPTED VARIABLES:**  X**_1_**, X**_1_^4^**, X**_2_^4^**, X**_3_**, X**_3_^4^**, X**_3_^5^**, X**_4_**, X**_4_^2^** | STOP THE PROCESS | **0.6385** |
